# Connectomics-based structural network alterations in obsessive-compulsive disorder

**DOI:** 10.1038/tp.2016.163

**Published:** 2016-09-06

**Authors:** T J Reess, O G Rus, R Schmidt, M A de Reus, M Zaudig, G Wagner, C Zimmer, M P van den Heuvel, K Koch

**Affiliations:** 1 Department of Neuroradiology & TUM-Neuroimaging Center (TUM-NIC), Klinikum rechts der Isar, Technische Universität München, Munich, Germany; 2 Graduate School of Systemic Neurosciences GSN, Ludwig-Maximilians-Universität, Biocenter, Munich, Germany; 3 Department of Neurology, Brain Center Rudolf Magnus, University Medical Center Utrecht, Utrecht, The Netherlands; 4 Department of Psychiatry, Brain Center Rudolf Magnus, University Medical Center Utrecht, Utrecht, The Netherlands; 5 Windach Institute and Hospital of Neurobehavioural Research and Therapy (WINTR), Windach, Germany; 6 Department of Psychiatry and Psychotherapy, Jena University Hospital, Jena, Germany

## Abstract

Given the strong involvement of affect in obsessive-compulsive disorder (OCD) and recent findings, the current cortico-striato-thalamo-cortical (CSTC) model of pathophysiology has repeatedly been questioned regarding the specific role of regions involved in emotion processing such as limbic areas. Employing a connectomics approach enables us to characterize structural connectivity on a whole-brain level, extending beyond the CSTC circuitry. Whole-brain structural networks of 41 patients and 42 matched healthy controls were analyzed based on 83 × 83 connectivity matrices derived from cortical and subcortical parcellation of structural T1-weighted magnetic resonance scans and deterministic fiber tracking based on diffusion tensor imaging data. To assess group differences in structural connectivity, the framework of network-based statistic (NBS) was applied. Graph theoretical measures were calculated to further assess local and global network characteristics. The NBS analysis revealed a single network consistently displaying decreased structural connectivity in patients comprising orbitofrontal, striatal, insula and temporo-limbic areas. In addition, graph theoretical measures indicated local alterations for amygdala and temporal pole while the overall topology of the network was preserved. To the best of our knowledge, this is the first study combining the NBS with graph theoretical measures in OCD. Along with regions commonly described in the CSTC model of pathophysiology, our results indicate an involvement of mainly temporo-limbic regions typically associated with emotion processing supporting their importance for neurobiological alterations in OCD.

## Introduction

Obsessive-compulsive disorder (OCD) is a psychiatric disorder characterized by recurrent, persistent and intrusive thoughts or images typically causing distress or anxiety (that is, obsessions), and repetitive behaviors aimed at reducing the feeling of anxiety (that is, compulsions).^1^ Traditionally, alterations in cortico-striato-thalamo-cortical (CSTC) circuitry have been associated with the pathophysiology of OCD.^2^ The CSTC model differentiates between affective and cognitive circuits, reflecting an impact of associated structures on emotional and cognitive functioning. However, it has recently been pointed out that the prevailing model does not specifically take into account the involvement of other structures such as amygdala and hippocampus and their interactions with frontal areas in mediating anxiety.^2^ Likewise, Menzies *et al.*^3^ concluded that several brain regions outside of the classical CSTC model may play a role in the pathophysiology. Based on a review of voxel-based morphometry (VBM) studies in OCD and in line with the aforementioned studies, Piras *et al.*^4^ similarly state an involvement of structural alterations in regions outside of the CSTC loops such as temporo-limbic regions to be relevant in OCD. Taken together, there is emerging evidence suggesting several brain regions other than fronto-thalamo-cortical areas to play a major role in the pathophysiology of OCD.

Progress has been made in identifying structural alterations in a broad range of psychiatric diseases using various methods such as VBM,^5^ and diffusion-weighted imaging.^6^ With the advent of connectomics, it is now feasible to shift the view from a regional perspective toward a network perspective based on the integration of various forms of anatomical data to assess connectivity of networks in brain disease,^7^ including psychiatric disorders.^8, 9, 10^ The conceptualization of the brain as a complex network calls for different approaches in modeling and analysis to infer information from brain magnetic resonance (MR) images and the mathematical framework of graph theory has proven to be especially useful in the analysis of such data.^11^ A broad range of measures can be calculated to assess topological properties of underlying brain graphs.^12^ Assessing these measures, one can potentially derive information about fundamental organizational properties in a specific group or compute differences between groups^13^ (for example, healthy controls vs psychiatric populations).^14^

To date, most studies addressing network alterations in OCD have focused on functional networks derived from resting-state functional magnetic resonance imaging (rs-fMRI).^15, 16, 17, 18^ Within a control network comprising frontal, parietal and cingulate cortex, as well as precuneus, thalamus and cerebellum, patients displayed alterations in small-world parameters.^15^ A recent study^18^ found decreased connectivity within the limbic system (amygdala and hippocampus) potentially related to problems with implicit learning and emotion processing observable in OCD patients. In addition, the same study reported an increase in connectivity within the executive/attention network in OCD possibly related to excessive monitoring and impairments in coping with threat/uncertainty. Only very few studies have examined alterations in structural networks in OCD using the method of connectomics. One study^19^ focused on cortical thickness and due to the nature of the specific measure had to disregard subcortical structures assumed to be of major importance in OCD. The only study^20^ defining structural networks based on diffusion data, reports disrupted topological organization in OCD as well as reduced nodal efficiency in frontal and parietal regions as well as the caudate.

An important question is whether functional alterations observed across studies have a structural correlate. Thus far, no study to date has focused on structural network alterations in OCD using/adopting a network-based statistic (NBS) approach.^14^ The current study aims at examining differences in the structural connectome in a fairly large sample of 41 OCD patients and 42 healthy controls based on the combination of anatomical and fiber tracking data derived from high-resolution structural MR scans and diffusion tensor imaging. Two approaches are used: NBS is applied to assess differences in specific topological features of networks, effectively controlling for the multiple comparison problem. Second, graph theoretical measures are applied to further identify potential changes in topologic properties. Since there is accumulating evidence for an involvement of regions outside the CSTC circuits in OCD, we expected to find structural alterations in areas not limited to CSTC loops. More specifically, due to the nature of the disease we expected an involvement of areas implicated in anxiety and emotion processing.

## Materials and methods

### Participants

A total of *n*=41 patients with OCD as the primary diagnosis according to DSM-IV criteria were included in the study. All diagnoses were made by an experienced psychiatrist from the Windach Institute and Hospital of Neurobehavioural Research and Therapy specialized in the treatment of OCD. As a control group *n*=42 age- and gender-matched healthy subjects were included (for demographic and clinical characteristics see Table 1).

Exclusion criteria for both groups were a history of clinically important head injuries, seizures or neurological diseases. There were no significant differences between healthy controls and OCD patients regarding age (*t*-test; *P*=0.73) and gender (*χ*^2^-test; *P*=0.42). At time of the study, *n*=12 patients were drug-naive or medication-free for at least 3 weeks. No patients were excluded due to comorbidities and *n*=22 patients had one or more comorbid diagnoses. Healthy controls with a history of psychiatric illness were excluded. All patients and controls were right-handed as assessed by Annett's handedness inventory.^21^ The patients were recruited from the Windach Institute and Hospital of Neurobehavioural Research and Therapy, Germany. To assess clinical severity of obsessive-compulsive symptoms, patients were handed the self-rated version of the Yale-Brown Obsessive-Compulsive Scale (Y-BOCS)^22, 23, 24^ as well as the Obsession-Compulsion Inventory revisited (OCI-R).^25, 26^ In addition, the Beck Depression Inventory (BDI)^27, 28^ was used to assess depressive symptoms. The study was approved by the local Ethics Committee of the Klinikum rechts der Isar, München.

### Image acquisition

MRI was conducted on a 3 T Philips Ingenia (Philips Healthcare, Best, the Netherlands) using a 12-channel (SENSE) head coil. Structural imaging consisted of a T1-weighted 3D MPRAGE sequence (170 slices, sagittal orientation, 240 × 240 matrix, 1mm isotropic resolution, TR=9 ms, TE=4 ms, flip angle=8°), and a diffusion-weighted imaging sequence (60 slices, 112 × 112 matrix, 2 mm isotropic resolution, TR=9000 ms, TE=57 ms, flip angle=90°, 32 diffusion directions, *b*-value=1000 s mm^−2^, two *b*=0 images).

### Image processing

Based on the high-resolution T1-weighted structural image, the cortical and subcortical structures as well as the brain stem were parcellated using Freesurfer (V5.1., http://surfer.nmr.mgh.harvard.edu/). Processing included automatic segmentation into gray and white matter tissue compartments followed by parcellation of the gray matter mask into distinct brain regions based on a normalized template. The resulting parcellation consisted of a total of 83 distinct brain regions of which 68 were cortical (34 per hemisphere), 14 subcortical (7 per hemisphere: thalamus, caudate, putamen, pallidum, hippocampus, amygdala, nucleus accumbens) and 1 represented the brainstem^29, 30, 31^ (see Supplementary Figure 1 for illustration of nodes). This parcellation scheme comprises several nodes that are thought to play an important role in the pathophysiology of OCD. Of special interest are several subcortical (for example, caudate, putamen, nucleus accumbens, amygdala, thalamus), as well as cortical regions (rostral middle frontal, medial orbital frontal, insula). In addition, this scheme is in line with studies examining other psychiatric diseases^8, 32^ and may thus facilitate comparison of results across diagnostic categories.

Diffusion data were corrected for movement and eddy-current distortions by realigning all diffusion-weighted images to the diffusion unweighted (*b*=0) scan. A tensor was fitted to each voxel's individual diffusion profile by applying a robust fitting method.^33^ Based on the fitted tensors, FA values as well as the preferred direction of diffusion (represented by the principal eigenvector) were calculated for each voxel.

### Tractography

Reconstruction of white matter tracts was based on Fiber Assignment by Continuous Tracking (FACT).^34, 35, 36^ To initiate tracking, eight seeds were placed in every voxel assigned to be white matter tissue based on the brain mask. Starting from each seed, tracking proceeded along the main diffusion direction propagating from voxel to voxel. Fiber tracking was terminated if the FA-value in a given voxel was <0.1, the angle between the preferred diffusion direction of two subsequent voxels exceeded 45° or the streamline exceeded the brain mask.

### Graph construction

A graph is the representation of a network in mathematical terms and is defined by a set of nodes, and a collection of edges describing the interactions between the nodes. To perform network analysis, a graph representing the structural connectivity network was constructed individually for each participant. Each node was assigned a specific brain region derived from the previous parcellation step. For every possible pair of nodes (N*_i_*,N*_j_*) it was determined whether a connection, that is a continuous streamline between N*_i_* and N*_j_*, was present or not. If present, the value of the connection was assigned the value of the number of streamlines (NOS) as indicated by the fiber tracking results. If a connection was absent, the connectivity value was set to zero. In this manner, for every participant, a single undirected, NOS-weighted graph was constructed. To avoid the influence of spurious connections, all edges with a streamline count of <2 were set to zero. A group threshold was applied to balance the influences of false-positive and false-negative reconstructions of tracts.^37^ In a first step, for each group separately, edges that were present in at least 60% of all group members were retained while all other edges were set to zero. In a second step, all edges that were present in at least 60% of the entire sample were retained. All subsequent analyses were conducted using the output from applying the 60% threshold within the entire sample. To check the stability of results, we additionally thresholded all matrices with varying thresholds, ranging from 30 to 90% with 5% increments and repeated all analysis (see Supplementary Tables 7 and 8).

### NBS analysis

Group differences between structural connectivity matrices were examined using the framework of the NBS introduced by Zalesky *et al.*^14^ NBS is a recently developed nonparametrical method to avoid the multiple comparison problem encountered when conducting mass univariate significance testing in graphs. Statistical significance is established for specific subsets of nodes that are mutually connected in topological rather than physical space. The first step in the analysis requires the calculation of a test statistic (here *t*-statistic) for each individual edge based on the differences in connectivity values (that is, NOS) between groups. Second, a primary component-forming threshold (here *P*<0.01, uncorrected) is applied to identify all edges displaying potential differences in connectivity strength. Third, all subthreshold edges are assessed for mutual connections forming clusters in topological space that may point toward the existence of non-chance clusters. Permutation testing is then applied to compute *P*-values for every component previously identified. To this end, steps 1 through 3 are repeated for each of the 5000 random permutations of group assignments (that is, patient or control), with noting the maximum cluster sizes of components resulting in a null distribution for the largest component size. The final hypothesis test is then carried out for the empirically determined components by comparing their sizes with the proportion of permutations yielding a component with equal or greater size. The final result controls the family-wise error rate at cluster level with *P*<0.05. Visualization of NBS networks was conducted using graphViz V2.3 (www.graphviz.org).^38^

### Graph theoretical analyses

All measures were calculated on the individual structural connectivity matrices using the Brain Connectivity Toolbox (http://www.brain-connectivity-toolbox.net/)^12^ under Matlab (R2014a, http://mathworks.com) and subsequently compared between groups. The following graph metrics were calculated for global description of the networks: (1) normalized global weighted clustering (*γ*), (2) normalized characteristic weighted path length (*λ*), (3) global strength, (4) total fiber counts. To calculate *γ* and *λ*, for every participant's brain network a set of 1000 random networks with identical degree sequence was formed. Subsequently for each of these networks, the weighted clustering coefficient and characteristic weighted path length were calculated and used for normalization. For description of nodal properties, the following node-specific (that is, region specific) graph metrics were calculated: (1) weighted clustering coefficient, (2) shortest weighted path length, (3) nodal strength. All comparisons involving graph measures were tested using permutation-based testing (10  000 permutations) corrected for multiple comparisons using false discovery rate (FDR)-correction^39^ if applicable.

### Analysis of nodal volumes

Volume differences on a nodal level can in principle lead to differences in the number of reconstructed streamlines and thus drive parts of the results. To check for such influences, a group comparison for the volumes of all nodes was conducted using permutation testing (10 000 permutations) and FDR-correction.

### Correlations

Potential relationships between clinical scores (Y-BOCS, OCI-R, BDI) and network measures were assessed including the NOS of edges comprising a significantly different cluster in the NBS analysis, as well as graph measures, displaying significant differences on a local and global scale. All correlations were corrected for multiple comparisons using FDR-correction.

### Influence of medication status

To assess the influence of medication status on results, we separately compared the subgroup of patients receiving medication with all healthy controls. This decision was based on the fact that the subsample of patients not receiving any medication (*n*=12) was likely to cause a lack of power in the statistical analysis. Instead, using the above mentioned approach, it was assessed whether the effects under consideration increased or decreased in magnitude, indicating a possible influence of medication status.

## Results

### NBS of structural connectivity alterations in OCD

**Figure 1:**
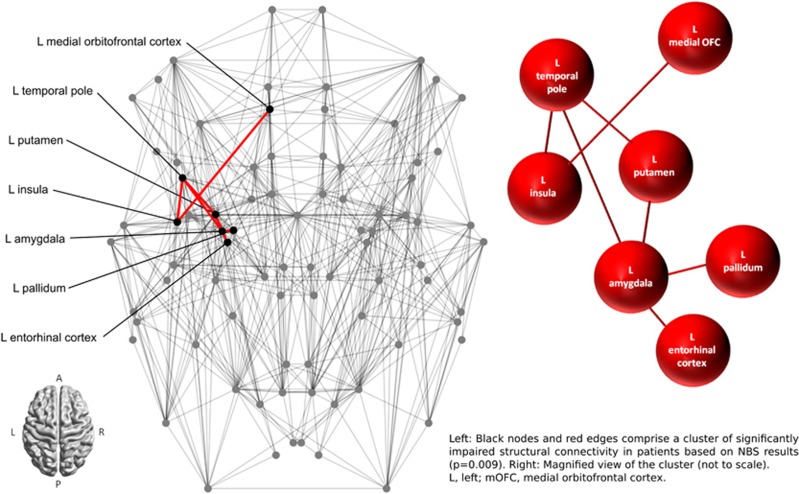
Connectome map representing nodes (circles) and edges (lines) of the structural network for the whole group. L, left; NBS, network-based statistic; OFC, orbitofrontal cortex.

**Figure 2:**
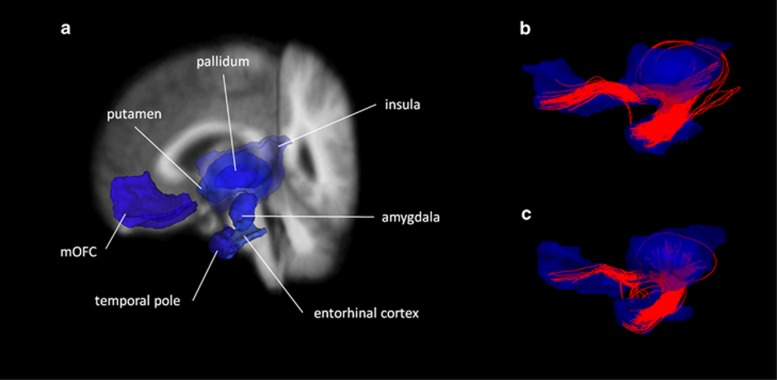
Illustration of the streamline trajectories comprising the edges of the significant NBS component. (**a**) For better anatomical reference, the nodes within the NBS component were extracted from the fsaverage segmentation and projected on the fsaverage anatomical T1-weighted image image. Fiber tracking results show aggregrated streamlines within the NBS component over all (**b**) controls and over all (**c**) patients. Aggregate fiber clouds have been downsampled to streamline counts representative of the subject groups. mOFC, medial orbitofrontal cortex; NBS, network-based statistic.

NBS analysis revealed a single network of decreased structural connectivity in OCD as compared with healthy controls (*P*=0.009). The network comprised a total of seven nodes connected by seven edges. The entire network was confined to the left hemisphere and included the following nodes: medial orbitofrontal cortex (mOFC), putamen, pallidum, amygdala, entorhinal cortex, insula and temporal pole (see Figure 1 for a depiction of the entire network structure). All connections between nodes were impaired in patients, that is, for each edge within the cluster, the NOS was consistently reduced in patients (see Table 2). For illustration purposes see Figure 2 depicting the aggregated streamline trajectories comprising the edges within the significantly impaired cluster for patients and healthy controls.

The analyses results obtained by varying the initial thresholds are presented in Supplementary Table 7. Overall, the NBS results were stable with only minor differences in cluster size for the most extreme thresholds (80–90%).

### Graph analysis

The overall topology of the networks was found to be in the small-world regime for both groups with the normalized global clustering coefficient *γ*>1 (mean±s.d.; patients: *γ*=3.0604±0.2456; controls: *γ*=3.0016±0.1324; *P*=0.183) and the normalized characteristic global path length *λ*~1 (patients: *λ*=1.2125±0.0891; controls: *λ*=1.2052±0.1085; *P*=0.748). There was a trend for a reduced global degree strength in patients (mean±s.d.; patients: 3986.2±771.0; controls: 4281.0±651.7; *P*=0.056), as well as a trend for a reduced total fiber count in patients (mean±s.d.; patients: 165 430±31 996; controls: 177 660±27 044; *P*=0.063). For local topological measures, the following significant differences were found: (1) decreased weighted clustering coefficients of left amygdala (*P*<0.001, FDR-corrected), left temporal pole (*P*<0.001, FDR-corrected) and right temporal pole (*P*=0.002, FDR-corrected); (2) increased shortest weighted path length of left amygdala (*P*<0.001, FDR-corrected), (3) decreased nodal strength of left amygdala (*P*<0.001, FDR-corrected). For nodes with significant differences based on uncorrected results see Supplementary Table 1. Regarding the stability of graph measure results, all results for amygdala are stable across the entire range of thresholds. There is some minor variation in significant differences for the weighted clustering coefficients and shortest weighted path lengths (see Supplementary Table 8). For a depiction of the global graph measures plotted as a function of connectivity matrix density see Supplementary Figure 2.

### Analysis of nodal volumes

Analysis of nodal volumes yielded no significant differences (*P*>0.05, FDR-corrected) for any of the nodes derived from cortical parcellation. See Supplementary Table 2 for details regarding the volume comparisons of all nodes comprising the NBS cluster.

### Correlation between clinical scores and connectivity parameters

There were no significant correlations between clinical scores and connections found to be impaired in the NBS analysis or for global and local graph measures (see Supplementary Tables 4–6 for reports of trend correlations between clinical scores and graph measures).

### Influence of medication status

The NBS results obtained from comparing healthy subjects with patients receiving medication yielded one significant cluster (*P*=0.047, corrected). It comprised a total of five nodes connected by five edges. The entire network was confined to the left hemisphere and included the following nodes: putamen, pallidum, amygdala, insula, and temporal pole. All connections between nodes were impaired in patients, that is, for each edge within the cluster, the NOS was consistently reduced in patients (see Supplementary Table 3).

For local topological measures, the following significant differences were found: (1) decreased weighted clustering coefficients of left amygdala (*P*<0.002) and left temporal pole (*P*<0.002); (2) increased shortest weighted path length of left amygdala (*P*<0.001), (3) decreased nodal strength of left amygdala (*P*<0.001).

## Discussion

The current study reports on NBS-based structural connectome differences between OCD patients and healthy controls, as well as graph theoretical analysis parameters. The NBS analysis revealed a single network with decreased structural connectivity in OCD. The affected subnetwork was lateralized to the left side and consisted of connections between mOFC, putamen, pallidum, amygdala, entorhinal cortex, insula and temporal pole. Several of these nodes are commonly implicated in the classical CSTC model of OCD such as the mOFC, putamen and pallidum providing evidence of the involvement of altered structural connectivity between these areas in the pathophysiology of OCD.

Interestingly, the connections between several nodes within the NBS cluster resemble a fronto-temporal pattern connecting mOFC, insula, temporal pole, amygdala and entorhinal cortex. Widespread anatomical connectivity between the aforementioned areas has been described in the literature. A major connection between the orbital and temporal gyrus is provided through the uncinate fasciculus (UF)^40^ which is commonly regarded as forming part of the limbic system due to connectivity and topology.^41^ Fibers are originating in the parahippocampal area, including the entorhinal cortex and temporal pole, reaching the orbital cortex after passing the amygdala and the limen insula.^41^ Some authors also describe an extension of the UF to the amygdala.^42^ Using a diffusion tensor imaging fiber tracking approach in humans, the anterior insula has been demonstrated to contain fibers connecting orbital/inferior frontal areas and temporal areas with parts of the fibers overlapping with the UF.^43^

Several studies using DWI measures and Tract-based Spatial Statistics (TBSS) have reported microstructural alterations within the UF in OCD, among others a decrease in FA values in left and right UF, as well as a reduced mean diffusivity in left UF in patients receiving medication^44^ and an increase in axial diffusivity in left and right UF in a pediatric sample of OCD patients.^45^ Regarding our results, a potential involvement of the UF seems possible as the connections between the aforementioned nodes are mainly provided through fibers that closely resemble the trajectory of the UF (also see Figure 2). Unlike TBSS, the connectomics approach taken in the current analysis does not focus on voxel-wise white matter alterations within a skeleton of the main fiber tracts but rather on the NOS between nodes. However, it is remarkable that there might be a convergence between results derived using various methods such as TBSS and NBS. Our results are also in line with a recent review/meta-analysis^46^ that comes to the conclusion that reductions in UF structural connectivity might be interpreted as the correlate of processing deficits in the emotional domain observed in neuropsychological research conducted with OCD patients.

A large body of evidence points toward wide-spread alterations in cortical volumes in OCD patients^4^ with changes mainly affecting frontal, temporal, thalamic and temporo-limbic areas. In addition, a recent multicenter study^47^ including over 400 patients, found a relative decrease of gray matter volume in the inferior frontal cortex extending to the anterior insula in OCD patients. As mentioned above, volume differences can lead to differences in the number of reconstructed streamlines and therefore influence results. The analysis of volume differences yielded no significant results between patients and controls for any of the nodes in the NBS cluster. This indicates that differences in the number of reconstructed streamlines are most likely not due to regional changes in volume but may rather indicate a correlate of underlying pathology. Note that the absence of volumetric differences in our sample does not necessarily contradict the findings from meta-analyses as they generally possess a higher statistical power to detect even subtle differences.

The importance of the amygdala is not specifically considered in the traditional CSTC model even though there is accumulating evidence indicating an involvement of this structure in the disease^48, 49, 50, 51, 52^ and an ongoing debate about its role in the pathophysiology of OCD.^2^ The NBS result clearly indicates an involvement of the amygdala. Specifically, within the impaired NBS cluster it is also the node displaying the highest binary degree (for example, the highest number of direct neighbors), providing a link between temporal and striatal areas. The important role is further underlined by results from graph theoretical measures indicating a decreased weighted clustering coefficient, a decrease in weighted degree strength, as well as an increased shortest path length for left amygdala. The clustering coefficient measures how strongly connected the neighbors of amygdala are and a decrease in clustering may thus point toward a decreased structural connectivity among the directly connected neighbors of the amygdala. This result might be interpreted such that in OCD, information normally traversing rather directly between immediate neighbors in healthy subjects may be more prone to be mediated via connections involving the amygdala, thus allowing it to exert an increased control over information flow between neighbors. As a measure of integration reflecting information about the connectivity between amygdala and all remaining nodes, the increase in shortest path length further indicates that amygdala is not as efficiently connected as in healthy controls. Taken together, there might be an additive effect in the sense that information is not only more prone to travel through amygdala, but also the connectivity between amygdala and its neighbors, as well as other brain regions is not as efficient.

Considering anxiety to be a core phenomenon of OCD, the finding of altered structural connectivity of limbic areas (such as OFC and amygdala) is especially striking since these areas are commonly thought to be central to emotion processing and behavioral regulation^53, 54^ with amygdala playing a central role for fear and anxiety. Hence, the alterations found in graph measures substantiate current discussions about the relevance of the amygdala for OCD and may represent the structural substrate of the pronounced feelings of anxiety preceding or accompanying patients' obsessive thoughts and compulsive actions.

Similar to left amygdala, both temporal poles also exhibited a decreased weighted clustering coefficient. The temporal pole has been implicated in various domains such as memory,^55^ as well as emotional processing, coupling sensory stimulation to emotional responses.^56, 57^ There is first evidence of an involvement of altered temporal pole structure and function in OCD. Van den Heuvel *et al.*^58^ found a negative correlation between checking symptoms and gray matter and white matter volume. Furthermore, the level of functional activation in the anterior temporal pole and amygdala during symptom provocation is reported to be associated with better subsequent treatment response to cognitive behavioral therapy.^59^ Taken together, previous and current findings provide support for the notion of structural alterations in amygdala and temporal pole in OCD which may be clinically relevant and may go along with an increase in functional activation. The association between increased functional activation in these limbic core regions and subsequent responsivity to treatment is in line with the emotional processing theory by Foa,^60^ which assumes that activation of limbic (and predominantly amygdala) regions during the experience of clinical symptoms is a prerequisite for successful exposure-based treatments for anxiety disorders. Whether there is a direct association between functional and structural alterations, as well as an analog association in OCD between structural alterations in these regions and individual treatment responsivity remains to be elucidated. To date there is only one study examining structural white matter network characteristics in OCD from a network perspective reporting several alterations in global and local graph measures.^20^ Their main findings are a reduction in global efficiency, as well as an increase in shortest path length, as well as *γ* and *λ* in patients. In addition, they report a significant correlation between *λ* and the Y-BOCS obsession score. There are, however, considerable methodological differences in comparison to our study that might have caused divergent findings. First and foremost, the composition of the sample differs regarding the number of patients (*n*=41 vs *n*=26), as well as other characteristics (with all patients being unmedicated with no comorbidities in the study by Zhong *et al.*^20^). Second, several parameters directly influencing the number of reconstructed streamlines differed substantially, such as the parcellation scheme which affects the volume of nodes and thus influences the number of voxels within each ROI to initialize tracking from. Furthermore, the tracking was initialized from one seed within each voxel in the study by Zhong *et al.* compared with eight seeds in the current study. In addition, we applied a more liberal threshold (FA-value<0.1 vs FA-value<0.2 used by Zhong *et al.*) as termination criteria for fiber tracking. Finally, we applied a 60% threshold to all connectivity matrices to find a good balance between false-positive and false-negative connections (see Materials and methods section). Taken together, the combination of differences in sample composition and choices influencing the number of reconstructed streamlines might explain divergent findings.

Apart from examinations of structural connectivity, there is an increasing number of studies using functional MRI to further elucidate the neurobiological basis of OCD. Göttlich *et al.*^18^ report a decrease in connectivity between limbic (amygdala, hippocampus and parahippocampal gyrus) and basal ganglia, as well as the default mode and executive/attention network in patients. In addition, the connectivity within the limbic network was reported to be impaired. Similarly, Jung *et al.*^61^ found an increased functional connectivity between nucleus accumbens and lateral orbitofrontal cortex during rest and a decrease in functional connectivity between nucleus accumbens and amygdala during incentive processing in patients. These results were interpreted as evidence in favor of abnormalities in modulatory influence of affective/motivational states on functional network connections in patients. Keeping in mind that the concept of functional connectivity is based on statistical associations and that the relationship between alterations in function and structure is not a straight-forward one-to-one mapping but rather complicated,^62^ there still seems to be an overlap between regions implicated in structural networks displaying alterations as shown in this study (amygdala, mOFC, striatal and temporal regions) and findings from altered functional connectivity between fronto-striato-temporal networks. It seems plausible that the structural alterations especially of connections between limbic regions might contribute to the proposed abnormalities in modulatory influence of affective/motivational states.

The current study has several limitations. Despite being fairly large, the examined sample comprised a certain proportion of patients with comorbid disorders, as well as a mix of medicated and unmedicated patients. Previous reports indicate an influence of selective serotonin reuptake inhibitor treatment on brain structure and function.^63, 64^ Nevertheless, the analysis of the subgroup of patients receiving medication is in good accordance with the primary analysis comparing healthy controls with all patients. The NBS analysis yielded one significant cluster that was only slightly varying in size. The magnitude of the differences in NOS-values increased for all edges in the NBS cluster of the medicated patients. This effect might be due to true differences in medication status. Alternatively, the connectivity differences could be related to differences in symptom severity. On average, the patient group receiving medication had a higher total Y-BOCS score than the unmedicated patients though formal statistical significance was not reached (medicated patients: 22.93±5.16 vs unmedicated patients: 19.83±3.16; *P*=0.095). The results of the graph measures computed for the subgroup of medicated patients were also rather similar to the results obtained from the original analysis with only the local clustering for the right temporal pole not reaching statistical significance. This again could be related to the above mentioned differences. Regarding the fact that selective serotonin reuptake inhibitors are the first line of treatment in OCD, influences of medication should be more rigorously assessed in future studies preferably comparing non-medicated and medicated groups with healthy controls separately.

Due to various limitations inherent to the method of fiber tracking, the accuracy of retrieved streamlines poses an issue in terms of false-positive and negative connections. To account for this fact, we applied a group threshold previously shown to strike a balance between erroneously assigning tracts.^37^ In the present study, a parcellation scheme commonly used in the Freesurfer suite was applied to increase comparability of results. Furthermore, the symptom heterogeneity typically found in OCD patients poses an issue. There is accumulating evidence that specific symptom dimensions in OCD can go along with specific alterations in neural processing, as well as structural alterations.^48, 58, 65^ Thus, it seems reasonable to explicitly consider the heterogeneity of symptom dimensions in future studies by trying to group patients according to symptom profile or predominant symptom dimension. Clearly, this approach would call for even bigger sample sizes to reach sufficient statistical power.

In summary, applying a network-based analysis strategy comparing structural brain networks of OCD patients and healthy controls we demonstrate impairments in a specific subnetwork in patients. Parts of the network overlap with regions commonly described in the CSTC model of the disease. However, several implicated regions and their connections are concentrated on a fronto-temporal axis indicating limbic structures to play a role in pathology.

## Figures and Tables

**Table 1:** Demographic and clinical sample characteristics

*Characteristics*	*OCD (*n=*41)*	*HC (*n=*42)*	P*-value*
	n *(%) or Mean±s.d. (range)*	n *(%) or Mean±s.d. (range)*	
Female	27 (65.9%)	24 (57.1%)	*P*=0.42
Age (Years)	32.5±10.0 (20–63)	31.8±8.3 (20–57)	*P*=0.73
Age of onset	15.9±6.40	—	
Disease duration	16.8±10.6	—	
*Y-BOCS total*	22.0±5.4 (15–36)	—	
Obsession	11.4±3.2 (4–17)	—	
Compulsions	10.6±3.5 (0–19)	—	
*OCI*-*R total*	25.2±9.2 (9–47)	—	
Hoarding	2.6±2.9 (0–11)	—	
Checking	5.9±3.2 (1–12)	—	
Ordering	3.5±3.7 (0–12)	—	
Neutralizing	1.9±2.6 (0–10)	—	
Washing	4.6±3.7 (0–11)	—	
Obsessing	6.8±3.0 (1–12)	—	
BDI	18.1±11.4 (0–53)	—	
*Comorbidities*	22 (53.7%)	—	
Depression	12 (29.3%)	—	
Anxiety	3 (7.3%)	—	
Depression and anxiety	3 (7.3%)	—	
Personality disorder	1 (2.4%)	—	
Eating disorder	1 (2.4%)	—	
Depression, anxiety, ADHD	1 (2.4%)	—	
Depression, eating disorder, personality disorder	1 (2.4%)	—	
*Medication*	29 (70.7%)	—	
SSRI	16 (55.2%)	—	
TCA	4 (13.8%)	—	
SSRI+antipsychotic	3 (10.3%)	—	
SSRNI	2 (6.9%)	—	
SSRI+methylphenidate	1 (3.4%)	—	
SSNRI+methylphenidate	1 (3.4%)	—	
NDRI+SSNRI	1 (3.4%)	—	
Benzodiazepine+antipsychotic	1 (3.4%)	—	

Abbreviations: ADHD, attention deficit hyperactivity disorder; BDI, Beck Depression Inventory; HC, healthy controls; NDRI, norepinephrine-dopamine reuptake inhibitor; OCD, obsessive-compulsive disorder; OCI-R, Obsession-Compulsion Inventory revisited; SSNRI, selective serotonin–norepinephrine reuptake inhibitor; SSRI, selective serotonin reuptake inhibitor; TCA, tricyclic antidepressant; Y-BOCS, Yale-Brown Obsessive-Compulsive Scale.

**Table 2:** Number of streamlines of edges comprising the network displaying significant group differences based on NBS analysis

*Network edges*	*NOS-value OCD mean±s.d.*	*NOS-value HC mean±s.d.*	P*-value/*t*-statistic*
L putamen–L amygdala	414.1±224.3	656.4±249.6	*P*<0.001, *t*=4.13
L pallidum–L amygdala	166.9±151.2	317.6±231.9	*P*<0.001, *t*=3.65
L putamen–L temporal pole	239.7±140.5	365.7±212.6	*P*=0.002, *t*=3.18
L amygdala–L temporal pole	393.0±169.0	533.0±246.7	*P*=0.003, *t*=3.01
L temporal pole–L insula	110.4±116.4	204.3±168.8	*P*=0.004, *t*=2.99
L mOFC–L insula	51.9±62.2	126.8±136.8	*P*=0.005, *t*=2.94
L amygdala–L entorhinal cortex	138.6±104.0	223.4±162.3	*P*=0.006, *t*=2.83

Abbreviations: HC, healthy controls; L, left; mOFC, medial orbitofrontal cortex; NBS, network-based statistic; NOS, number of streamlines; OCD, obsessive-compulsive disorder.

Mean±s.d. for the number of streamlines for each edge within the NBS cluster.

## References

[bib1] American Psychiatric ADiagnostic and Statistical Manual of Mental Disorders. Text Revision (DSM-IV-TR). American Psychiatric Association: : Washington, DC, 2000.

[bib2] Milad MR, Rauch SL. Obsessive-compulsive disorder: beyond segregated cortico-striatal pathways. Trends Cogn Sci 2012; 16: 43–51. 22138231 10.1016/j.tics.2011.11.003 PMC4955838

[bib3] Menzies L, Chamberlain SR, Laird AR, Thelen SM, Sahakian BJ, Bullmore ET. Integrating evidence from neuroimaging and neuropsychological studies of obsessive-compulsive disorder: the orbitofronto-striatal model revisited. Neurosci Biobehav Rev 2008; 32: 525–549. 18061263 10.1016/j.neubiorev.2007.09.005 PMC2889493

[bib4] Piras F, Piras F, Chiapponi C, Girardi P, Caltagirone C, Spalletta G. Widespread structural brain changes in OCD: a systematic review of voxel-based morphometry studies. Cortex 2015; 62: 89–108. 23582297 10.1016/j.cortex.2013.01.016

[bib5] Goodkind M, Eickhoff SB, Oathes DJ, Jiang Y, Chang A, Jones-Hagata LB et al. Identification of a common neurobiological substrate for mental illness. JAMA Psychiatry 2015; 72: 305–315. 25651064 10.1001/jamapsychiatry.2014.2206 PMC4791058

[bib6] White T, Nelson M, Lim KO. Diffusion tensor imaging in psychiatric disorders. Top Magn Reson Imaging 2008; 19: 97–109. 19363432 10.1097/RMR.0b013e3181809f1e

[bib7] Griffa A, Baumann PS, Thiran JP, Hagmann P. Structural connectomics in brain diseases. Neuroimage 2013; 80: 515–526. 23623973 10.1016/j.neuroimage.2013.04.056

[bib8] Korgaonkar MS, Fornito A, Williams LM, Grieve SM. Abnormal structural networks characterize major depressive disorder: a connectome analysis. Biol Psychiatry 2014; 76: 567–574. 24690111 10.1016/j.biopsych.2014.02.018

[bib9] van den Heuvel MP, Mandl RC, Stam CJ, Kahn RS, Hulshoff Pol HE. Aberrant frontal and temporal complex network structure in schizophrenia: a graph theoretical analysis. J Neurosci 2010; 30: 15915–15926. 21106830 10.1523/JNEUROSCI.2874-10.2010 PMC6633761

[bib10] Narr KL. Leaver AM. Connectome and schizophrenia. Curr Opin Psychiatry 2015; 28: 229–235. 25768086 10.1097/YCO.0000000000000157

[bib11] Bullmore ET, Bassett DS. Brain graphs: graphical models of the human brain connectome. Annu Rev Clin Psychol 2011; 7: 113–140. 21128784 10.1146/annurev-clinpsy-040510-143934

[bib12] Rubinov M, Sporns O. Complex network measures of brain connectivity: uses and interpretations. Neuroimage 2010; 52: 1059–1069. 19819337 10.1016/j.neuroimage.2009.10.003

[bib13] Fornito A, Zalesky A, Breakspear M. Graph analysis of the human connectome: promise, progress, and pitfalls. Neuroimage 2013; 80: 426–444. 23643999 10.1016/j.neuroimage.2013.04.087

[bib14] Zalesky A, Fornito A, Bullmore ET. Network-based statistic: identifying differences in brain networks. Neuroimage 2010; 53: 1197–1207. 20600983 10.1016/j.neuroimage.2010.06.041

[bib15] Zhang T, Wang J, Yang Y, Wu Q, Li B, Chen L et al. Abnormal small-world architecture of top-down control networks in obsessive-compulsive disorder. J Psychiatry Neurosci 2011; 36: 23–31. 20964957 10.1503/jpn.100006 PMC3004972

[bib16] Shin DJ, Jung WH, He Y, Wang J, Shim G, Byun MS et al. The effects of pharmacological treatment on functional brain connectome in obsessive-compulsive disorder. Biol Psychiatry 2014; 75: 606–614. 24099506 10.1016/j.biopsych.2013.09.002

[bib17] Hou JM, Zhao M, Zhang W, Song LH, Wu WJ, Wang J et al. Resting-state functional connectivity abnormalities in patients with obsessive-compulsive disorder and their healthy first-degree relatives. J Psychiatry Neurosci 2014; 39: 304–311. 24866415 10.1503/jpn.130220 PMC4160359

[bib18] Gottlich M, Kramer UM, Kordon A, Hohagen F, Zurowski B. Decreased limbic and increased fronto-parietal connectivity in unmedicated patients with obsessive-compulsive disorder. Hum Brain Mapp 2014; 35: 5617–5632. 25044747 10.1002/hbm.22574 PMC6868939

[bib19] Kim SG, Jung WH, Kim SN, Jang JH, Kwon JS. Disparity between dorsal and ventral networks in patients with obsessive-compulsive disorder: evidence revealed by graph theoretical analysis based on cortical thickness from MRI. Front Hum Neurosci 2013; 7: 302. 23840184 10.3389/fnhum.2013.00302 PMC3699763

[bib20] Zhong Z, Zhao T, Luo J, Guo Z, Guo M, Li P et al. Abnormal topological organization in white matter structural networks revealed by diffusion tensor tractography in unmedicated patients with obsessive-compulsive disorder. Prog Neuropsychopharmacol Biol Psychiatry 2014; 51: 39–50. 24440373 10.1016/j.pnpbp.2014.01.005

[bib21] Annett M. A classification of hand preference by association analysis. Br J Psychol 1970; 61: 303–321. 5457503 10.1111/j.2044-8295.1970.tb01248.x

[bib22] Goodman WK, Price LH, Rasmussen SA, Mazure C, Fleischmann RL, Hill CL et al. The Yale-Brown Obsessive Compulsive Scale. I. Development, use, and reliability. Arch Gen Psychiatry 1989; 46: 1006–1011. 2684084 10.1001/archpsyc.1989.01810110048007

[bib23] Hand I, Büttner-Westphal H. Die Yale-Brown Obsessive Compulsive Scale (Y-BOCS): ein halbstrukturiertes interview zur beurteilung des schweregrades von denk- und handlungszwängen. Verhaltenstherapie 1991; 1: 223–225.

[bib24] Steketee G, Frost R, Bogart K. The Yale-Brown Obsessive Compulsive Scale: interview versus self-report. Behav Res Ther 1996; 34: 675–684. 8870295 10.1016/0005-7967(96)00036-8

[bib25] Foa EB, Huppert JD, Leiberg S, Langner R, Kichic R, Hajcak G et al. The obsessive-compulsive inventory: development and validation of a short version. Psychol Assess 2002; 14: 485–496. 12501574

[bib26] Gonner S, Leonhart R, Ecker W. The Obsessive-Compulsive Inventory-Revised (OCI-R): validation of the German version in a sample of patients with OCD, anxiety disorders, and depressive disorders. J Anxiety Disord 2008; 22: 734–749. 17913454 10.1016/j.janxdis.2007.07.007

[bib27] Beck AT, Steer RA, Brown G. Manual for the Beck Depression Inventory-II. Psychological Corporation: San Antonio, TX, USA, 1996.

[bib28] Hautzinger M, Keller F, Kühner C, BDI-II. Beck-Depressions-Inventar Revision. 2. Auflage (ed.). Pearson Assessment: Frankfurt am Main, Germany, 2009.

[bib29] Fischl B, Sereno MI, Dale AM. Cortical surface-based analysis. II: Inflation, flattening, and a surface-based coordinate system. Neuroimage 1999; 9: 195–207. 9931269 10.1006/nimg.1998.0396

[bib30] Fischl B, van der Kouwe A, Destrieux C, Halgren E, Segonne F, Salat DH et al. Automatically parcellating the human cerebral cortex. Cereb Cortex 2004; 14: 11–22. 14654453 10.1093/cercor/bhg087

[bib31] Desikan RS, Segonne F, Fischl B, Quinn BT, Dickerson BC, Blacker D et al. An automated labeling system for subdividing the human cerebral cortex on MRI scans into gyral based regions of interest. Neuroimage 2006; 31: 968–980. 16530430 10.1016/j.neuroimage.2006.01.021

[bib32] van den Heuvel MP, Sporns O, Collin G, Scheewe T, Mandl RC, Cahn W et al. Abnormal rich club organization and functional brain dynamics in schizophrenia. JAMA Psychiatry 2013; 70: 783–792. 23739835 10.1001/jamapsychiatry.2013.1328

[bib33] Chang LC, Jones DK, Pierpaoli C. RESTORE: robust estimation of tensors by outlier rejection. Magn Reson Med 2005; 53: 1088–1095. 15844157 10.1002/mrm.20426

[bib34] Mori S, van Zijl PC. Fiber tracking: principles and strategies—a technical review. NMR Biomed 2002; 15: 468–480. 12489096 10.1002/nbm.781

[bib35] Mori S, Crain BJ, Chacko VP, van Zijl PC. Three-dimensional tracking of axonal projections in the brain by magnetic resonance imaging. Ann Neurol 1999; 45: 265–269. 9989633 10.1002/1531-8249(199902)45:2<265::aid-ana21>3.0.co;2-3

[bib36] Mori S, Kaufmann WE, Davatzikos C, Stieltjes B, Amodei L, Fredericksen K et al. Imaging cortical association tracts in the human brain using diffusion-tensor-based axonal tracking. Magn Reson Med 2002; 47: 215–223. 11810663 10.1002/mrm.10074

[bib37] de Reus MA, van den Heuvel MP. Estimating false positives and negatives in brain networks. Neuroimage 2013; 70: 402–409. 23296185 10.1016/j.neuroimage.2012.12.066

[bib38] Gansner ER, North SC. An open graph visualization system and its applications to software engineering. Software Pract Exper 2000; 30: 1203–1233.

[bib39] Benjamini Y, Hochberg Y. Controlling the false discovery rate - a practical and powerful approach to multiple testing. J R Stat Soc Ser B Stat Methodol 1995; 57: 289–300.

[bib40] Ebeling U, von Cramon D. Topography of the uncinate fascicle and adjacent temporal fiber tracts. Acta Neurochir (Wien) 1992; 115: 143–148. 1605083 10.1007/BF01406373

[bib41] Von Der Heide RJ, Skipper LM, Klobusicky E, Olson IR. Dissecting the uncinate fasciculus: disorders, controversies and a hypothesis. Brain 2013; 136: 1692–1707. 23649697 10.1093/brain/awt094 PMC3673595

[bib42] Thiebaut de Schotten M, Dell'Acqua F, Valabregue R, Catani M. Monkey to human comparative anatomy of the frontal lobe association tracts. Cortex 2012; 48: 82–96. 22088488 10.1016/j.cortex.2011.10.001

[bib43] Cloutman LL, Binney RJ, Drakesmith M, Parker GJ, Lambon Ralph MA. The variation of function across the human insula mirrors its patterns of structural connectivity: evidence from *in vivo* probabilistic tractography. Neuroimage 2012; 59: 3514–3521. 22100771 10.1016/j.neuroimage.2011.11.016

[bib44] Benedetti F, Giacosa C, Radaelli D, Poletti S, Pozzi E, Dallaspezia S et al. Widespread changes of white matter microstructure in obsessive-compulsive disorder: effect of drug status. Eur Neuropsychopharmacol 2013; 23: 581–593. 22954900 10.1016/j.euroneuro.2012.07.002

[bib45] Jayarajan RN, Venkatasubramanian G, Viswanath B, Janardhan Reddy YC, Srinath S, Vasudev MK et al. White matter abnormalities in children and adolescents with obsessive-compulsive disorder: a diffusion tensor imaging study. Depress Anxiety 2012; 29: 780–788. 22323419 10.1002/da.21890

[bib46] Piras F, Piras F, Caltagirone C, Spalletta G. Brain circuitries of obsessive compulsive disorder: a systematic review and meta-analysis of diffusion tensor imaging studies. Neurosci Biobehav Rev 2013; 37: 2856–2877. 24177038 10.1016/j.neubiorev.2013.10.008

[bib47] de Wit SJ, Alonso P, Schweren L, Mataix-Cols D, Lochner C, Menchon JM et al. Multicenter voxel-based morphometry mega-analysis of structural brain scans in obsessive-compulsive disorder. Am J Psychiatry 2014; 171: 340–349. 24220667 10.1176/appi.ajp.2013.13040574

[bib48] Via E, Cardoner N, Pujol J, Alonso P, Lopez-Sola M, Real E et al. Amygdala activation and symptom dimensions in obsessive-compulsive disorder. Br J Psychiatry 2014; 204: 61–68. 24262816 10.1192/bjp.bp.112.123364

[bib49] Simon D, Adler N, Kaufmann C, Kathmann N. Amygdala hyperactivation during symptom provocation in obsessive-compulsive disorder and its modulation by distraction. Neuroimage Clin 2014; 4: 549–557. 24818080 10.1016/j.nicl.2014.03.011 PMC3984443

[bib50] Cardoner N, Harrison BJ, Pujol J, Soriano-Mas C, Hernandez-Ribas R, Lopez-Sola M et al. Enhanced brain responsiveness during active emotional face processing in obsessive compulsive disorder. World J Biol Psychiatry 2011; 12: 349–363. 21781000 10.3109/15622975.2011.559268

[bib51] Simon D, Kaufmann C, Musch K, Kischkel E, Kathmann N. Fronto-striato-limbic hyperactivation in obsessive-compulsive disorder during individually tailored symptom provocation. Psychophysiology 2010; 47: 728–738. 20158678 10.1111/j.1469-8986.2010.00980.x

[bib52] van Velzen LS, de Wit SJ, Curcic-Blake B, Cath DC, van Vries FE, Veltman DJ et al. Altered inhibition-related frontolimbic connectivity in obsessive-compulsive disorder. Hum Brain Mapp 2015; 36: 4064–4075. 26183689 10.1002/hbm.22898 PMC6869170

[bib53] Phelps EA, LeDoux JE. Contributions of the amygdala to emotion processing: from animal models to human behavior. Neuron 2005; 48: 175–187. 16242399 10.1016/j.neuron.2005.09.025

[bib54] Dolan RJ. The human amygdala and orbital prefrontal cortex in behavioural regulation. Philos Trans R Soc Lond B Biol Sci 2007; 362: 787–799. 17403643 10.1098/rstb.2007.2088 PMC2429997

[bib55] Simmons WK, Martin A. The anterior temporal lobes and the functional architecture of semantic memory. J Int Neuropsychol Soc 2009; 15: 645–649. 19631024 10.1017/S1355617709990348 PMC2791360

[bib56] Olson IR, Plotzker A, Ezzyat Y. The Enigmatic temporal pole: a review of findings on social and emotional processing. Brain 2007; 130: 1718–1731. 17392317 10.1093/brain/awm052

[bib57] Blaizot X, Mansilla F, Insausti AM, Constans JM, Salinas-Alaman A, Pro-Sistiaga P et al. The human parahippocampal region: I. Temporal pole cytoarchitectonic and MRI correlation. Cereb Cortex 2010; 20: 2198–2212. 20064939 10.1093/cercor/bhp289 PMC2923216

[bib58] van den Heuvel OA, Remijnse PL, Mataix-Cols D, Vrenken H, Groenewegen HJ, Uylings HB et al. The major symptom dimensions of obsessive-compulsive disorder are mediated by partially distinct neural systems. Brain 2009; 132: 853–868. 18952675 10.1093/brain/awn267

[bib59] Olatunji BO, Ferreira-Garcia R, Caseras X, Fullana MA, Wooderson S, Speckens A et al. Predicting response to cognitive behavioral therapy in contamination-based obsessive-compulsive disorder from functional magnetic resonance imaging. Psychol Med 2014; 44: 2125–2137. 24229474 10.1017/S0033291713002766

[bib60] Foa EB, McNally RJ. Mechanisms of change in exposure therapy. In:Rapee RM (ed). Current Controversies in the Anxiety Disorders. Guilford Press: New York, NY, USA, 1996, pp 329–343.

[bib61] Jung WH, Kang DH, Kim E, Shin KS, Jang JH, Kwon JS. Abnormal corticostriatal-limbic functional connectivity in obsessive-compulsive disorder during reward processing and resting-state. Neuroimage Clin 2013; 3: 27–38. 24179846 10.1016/j.nicl.2013.06.013 PMC3791288

[bib62] Wang Z, Dai Z, Gong G, Zhou C, He Y. Understanding structural-functional relationships in the human brain: a large-scale network perspective. Neuroscientist 2015; 21: 290–305. 24962094 10.1177/1073858414537560

[bib63] Hoexter MQ, de Souza Duran FL, D'Alcante CC, Dougherty DD, Shavitt RG, Lopes AC et al. Gray matter volumes in obsessive-compulsive disorder before and after fluoxetine or cognitive-behavior therapy: a randomized clinical trial. Neuropsychopharmacology 2012; 37: 734–745. 22030709 10.1038/npp.2011.250 PMC3260985

[bib64] Kraus C, Ganger S, Losak J, Hahn A, Savli M, Kranz GS et al. Gray matter and intrinsic network changes in the posterior cingulate cortex after selective serotonin reuptake inhibitor intake. Neuroimage 2014; 84: 236–244. 23988273 10.1016/j.neuroimage.2013.08.036

[bib65] Harrison BJ, Pujol J, Cardoner N, Deus J, Alonso P, Lopez-Sola M et al. Brain corticostriatal systems and the major clinical symptom dimensions of obsessive-compulsive disorder. Biol Psychiatry 2013; 73: 321–328. 23200527 10.1016/j.biopsych.2012.10.006

