# Supplementary material for: Connectomics-based structural network alterations in obsessive-compulsive disorder
Source: Transl Psychiatry. 2016 Sep 6;6(9):e882–. doi: 10.1038/tp.2016.163 (PMC5048203; doi:10.1038/tp.2016.163)

Supplementary Information

Reess TJ, Rus OG, Schmidt R, de Reus MA, Zaudig M, Wagner G, Zimmer C, van den Heuvel MP, Koch K.
Connectomics-based structural network alterations in obsessive-compulsive disorder.

Supplementary Table 1. Group differences for local graph measures significant at a level of $p<0.05$, uncorrected.

Supplementary Table 2. Group comparison of volumes (mm^3) for nodes comprising the NBS network.

Supplementary Table 3. Number of streamlines of edges comprising the network displaying significant group differences based on NBS analysis of only medicated patients (n=29).

Supplementary Table 4. Correlations between significantly impaired edges from the NBS cluster and clinical scores.

Supplementary Table 5. Correlations between total degree strength and clinical scores.

Supplementary Table 6. Correlations between significantly different local graph measures and clinical scores.

Supplementary Table 7. NBS results of analysis conducted with various group-thresholds.

Supplementary Table 8. Results for differences in local graph measures and global degree strength computed for various group thresholds.

Supplementary Figure 1. Illustration of the parcellation scheme used for defining the nodes.

Supplementary Figure 2. Global graph measures and small-worldness plotted as a function of connectivity matrix densities.

Supplementary Table 1. Group differences for local graph measures significant at a level of $p<0.05$, uncorrected.

**Weigthed Connectivity Strength**	**p-value**	**Weighted Clustering Coefficient**	**p-value**
Subcortical structures		*Subcortical structures*	
L pallidum	p = 0.032; HC > OCD	R putamen	p = 0.022; HC > OCD
R putamen	p = 0.021; HC > OCD	R n. accumbens	p = 0.024; HC > OCD
R pallidum	p = 0.002; HC > OCD		
R n. accumbens	p = 0.005; HC > OCD		
Cortical structures		*Cortical structures*	
L entorhinal cortex	p = 0.016; HC > OCD	L hippocampus	p = 0.043; HC > OCD
L pericalcarine	p = 0.024; OCD > HC	L entorhinal cortex	P = 0.019; HC > OCD
L precuneus	p = 0.046; HC > OCD		
L temporal pole	p = 0.007; HC > OCD		
R entorhinal cortex	p = 0.041; HC > OCD		
R rostral middle frontal cortex	p = 0.011; HC > OCD		
R superior parietal cortex	p = 0.027; HC > OCD		
R temporal pole	p = 0.040; HC > OCD		
R insula	p = 0.041; HC > OCD		
brain stem	p = 0.013; HC > OCD		

HC, healthy controls; OCD, obsessive-compulsive disorder; L, left; R, right; all tests are permutation based with 10000 permutations

Supplementary Table 1 (continued)

Shortest Path Length	p-value	Shortest Path Length	p-value
Subcortical structures		*Subcortical structures*	
L thalamus	p = 0.007; HC < OCD	R caudate	p = 0.039; HC < OCD
L caudate	p = 0.030; HC < OCD	R putamen	p = 0.018; HC < OCD
L putamen	p = 0.040; HC < OCD	R pallidum	p = 0.008; HC < OCD
L pallidum	p = 0.008; HC < OCD	R amygdala	p = 0.018; HC < OCD
L hippocampus	p = 0.021; HC < OCD	R n. accumbens	p = 0.037; HC < OCD
Cortical structures		*Cortical structures*	
L entorhinal cortex	p = 0.001; HC < OCD	R postcentral	p = 0.032; HC < OCD
L isthmuscingulate cortex	p = 0.043; HC < OCD	R precentral	p = 0.020; HC < OCD
L superior parietal cortex	p = 0.021; HC < OCD	R precuneus	p = 0.047; HC < OCD
L temporal pole	p = 0.006; HC < OCD	R rostral middle frontal cortex	p = 0.012; HC < OCD
		R superior parietal cortex	p = 0.015; HC < OCD
		R supramarginal gyrus	p = 0.040; HC < OCD
		R temporal pole	p = 0.008; HC < OCD
		R transversetemporal	p = 0.031; HC < OCD
Brain stem	p = 0.020; HC < OCD		

HC, healthy controls; OCD, obsessive-compulsive disorder; L, left; R, right; all tests are permutation based with 10000 permutations

Supplementary Table 2. Group comparison of volumes (mm^3) for nodes comprising the NBS network.

**Node**	**Volume (mm^3) OCD** mean ± SD	**Volume (mm^3) HC** mean ± SD	**p-value**	**Cohen's d [95% - confidence interval]**
L putamen	6562.1 ± 809.9	6436.1 ± 765.9	p = 0.476	- 0.16 [-0.59 – 0.27]
L pallidum	1736.0 ± 233.7	1804.6 ± 231.8	p = 0.192	0.30 [-0.14 – 0.73]
L temporal pole	2656.1 ± 397.0	2702.3 ± 433.1	p = 0.594	0.11 [-0.32 – 0.54]
L insula	6484.3 ± 779.6	6633.9 ± 809.3	p = 0.408	0.19 [-0.24 – 0.62]
L amygdala	1730.2 ± 251.2	1786.6 ± 228.9	p = 0.292	0.24 [-0.20 – 0.67]
L mOFC	4555.0 ± 647.0	4698.4 ± 767.9	p = 0.357	0.20 [-0.23 – 0.63]
L entorhinal cortex	1807.8 ± 297.8	1908.6 ± 357.4	p = 0.168	0.31 [-0.13 – 0.74]

HC, healthy controls; OCD, obsessive-compulsive disorder; L, left; mOFC, medial orbitofrontal cortex; SD, standard deviation

Supplementary Table 3. Number of streamlines of edges comprising the network displaying significant group differences based on NBS analysis of only medicated patients (n=29).

**Network edges**	**NOS-value $OCD_{medicated}$ Mean ± SD**	**NOS-value HC Mean ± SD**	**p-value / t-statistic**
L putamen – L amygdala	440.2 ± 232.9	656.4 ± 249.6	p < 0.001, t = 3.76
L pallidum – L amygdala	173.5 ± 204.3	317.6 ± 231.9	p = 0.003, t = 3.14
L putamen – L temporal pole	236.3 ± 186.8	365.7 ± 212.6	p = 0.006, t = 2.87
L amygdala – L temporal pole	384.3 ± 211.4	533.0 ± 246.7	p = 0.005, t = 2.91
L temporal pole – L insula	109.1 ± 148.1	204.3 ± 168.8	p = 0.008, t = 2.76

Mean ± standard deviation for the number of streamlines for each edge within the NBS cluster. NOS, number of streamlines; L, left; SD, standard deviation

Supplementary Table 4. Correlations between significantly impaired edges from the NBS cluster and clinical scores. Only trend correlations (p < 0.1) are reported.

**Network Eedges (NOS)**	**clinical score / demographic information**	**p-value / pearson correlation coefficient**
L putamen – L amygdala	age of onset	p = 0.079, r = 0.288
L pallidum – L amygdala	washing (OCI-R)	p = 0.080, r = -0.284
L putamen – L temporal pole	obsessing (OCI-R)	p = 0.059, r = -0.297
	obsession (Y-BOCS)	p = 0.087, r = -0.270
	total (Y-BOCS)	p = 0.091, r = -0.268

NOS: number of streamlines; All reported p-values are uncorrected

Supplementary Table 5. Correlations between total degree strength and clinical scores. Only trend correlations ($p < 0.1$) are reported.

Global Graph Measures	clinical score / demographic information	p-value, correlation coefficient
Global degree strength	disease duration	p = 0.004, r = -0.443*<br>p = 0.416, r = -0.132‡

* pearson correlation coefficient; ‡ partial correlation coefficient [controlling for age]

Supplementary Table 6. Correlations between significantly different local graph measures and clinical scores. Only trend correlations ($p < 0.1$) are reported.

Local Graph Measures	clinical score / demographic information	p-value, correlation coefficient
Amygdala – clustering	obsessing (OCI-R)	p = 0.082, r = 0.275*
Amygdala – shortest path length	disease duration	p = 0.055, r = 0.302*<br>p = 0.397, r = -0.138‡
R temporal pole – clustering	hoarding (OCI-R)	p = 0.016, r = -0.374*
	checking (OCI-R)	p = 0.056, r = 0.301*
	washing (OCI-R)	p = 0.050, r = -0.308*

* pearson correlation coefficient; ‡ partial correlation coefficient [controlling for age]

Supplementary Table 7. NBS results of analysis conducted with various group-thresholds.

% threshold	density (%)	cluster size	p-value
30	25.51 %	7	0.019
35	23.63 %	7	0.016
40	22.51 %	7	0.015
45	20.86 %	7	0.014
50	19.54 %	7	0.013
55	17.95 %	7	0.010
60	17.34 %	7	0.009
65	14.96 %	7	0.009
70	13.69 %	7	0.005
75	11.99 %	7	0.005
80	10.11 %	6	0.008
85	8.96 %	6	0.006
90	7.49 %	6	0.005

All NBS analyses were conducted with 5000 permutations

The significantly impaired cluster for the NBS analysis is essentially stable for the various thresholds. Only for the 80%, 85%, and 90% threshold the cluster size is reduced by one with the connection between mOFC and insula not being part of the cluster.

Supplementary Table 8. Results for differences in local graph measures and global degree strength computed for various group thresholds.

% threshold	weighted clustering	Shortest weighted path	nodal strength	Global degree strength	density (in %)
30	no differences	L amygdala	L amygdala	p = 0.081	25.51 %
35	no differences	L amygdala<br>L entorhinal	L amygdala	p = 0.080	23.63 %
40	L amygdala	L amygdala<br>L entorhinal	L amygdala	p = 0.080	22.51 %
45	L amygdala	L amygdala<br>L entorhinal	L amygdala	p = 0.080	20.86 %
50	L amygdala	L amygdala	L amygdala	p = 0.070	19.54 %
55	L amygdala<br>L temporal pole	L amygdala<br>L entorhinal	L amygdala	p = 0.063	17.95 %
60	L amygdala<br>L temporal pole<br>R temporal pole	L amygdala	L amygdala	p = 0.056	17.34 %
65	L amygdala<br>L temporal pole<br>R temporal pole	L amygdala<br>L entorhinal	L amygdala	p = 0.054	14.96 %
70	L amygdala<br>L temporal pole<br>R temporal pole	L amygdala<br>L entorhinal	L amygdala	p = 0.047	13.69 %
75	L amygdala<br>L temporal pole	L amygdala<br>L entorhinal	L amygdala	p = 0.044	11.99 %
80	L amygdala<br>L temporal pole	L amygdala<br>L entorhinal	L amygdala<br>L temporal pole	p = 0.051	10.11 %
85	L amygdala<br>L temporal pole	L amygdala<br>L entorhinal	L amygdala<br>L temporal pole	p = 0.045	8.96 %
90	L amygdala<br>L temporal pole<br>R temporal pole	L amygdala	L amygdala	P = 0.034	7.49 %

all tests are permutation based with 10000 permutations

All reported nodes were significantly different regarding the graph measure in question, based on FDR-corrected, permutation based testing with 5000 permutations

Supplementary Figure 1. Illustration of the parcellation scheme used for defining the nodes.

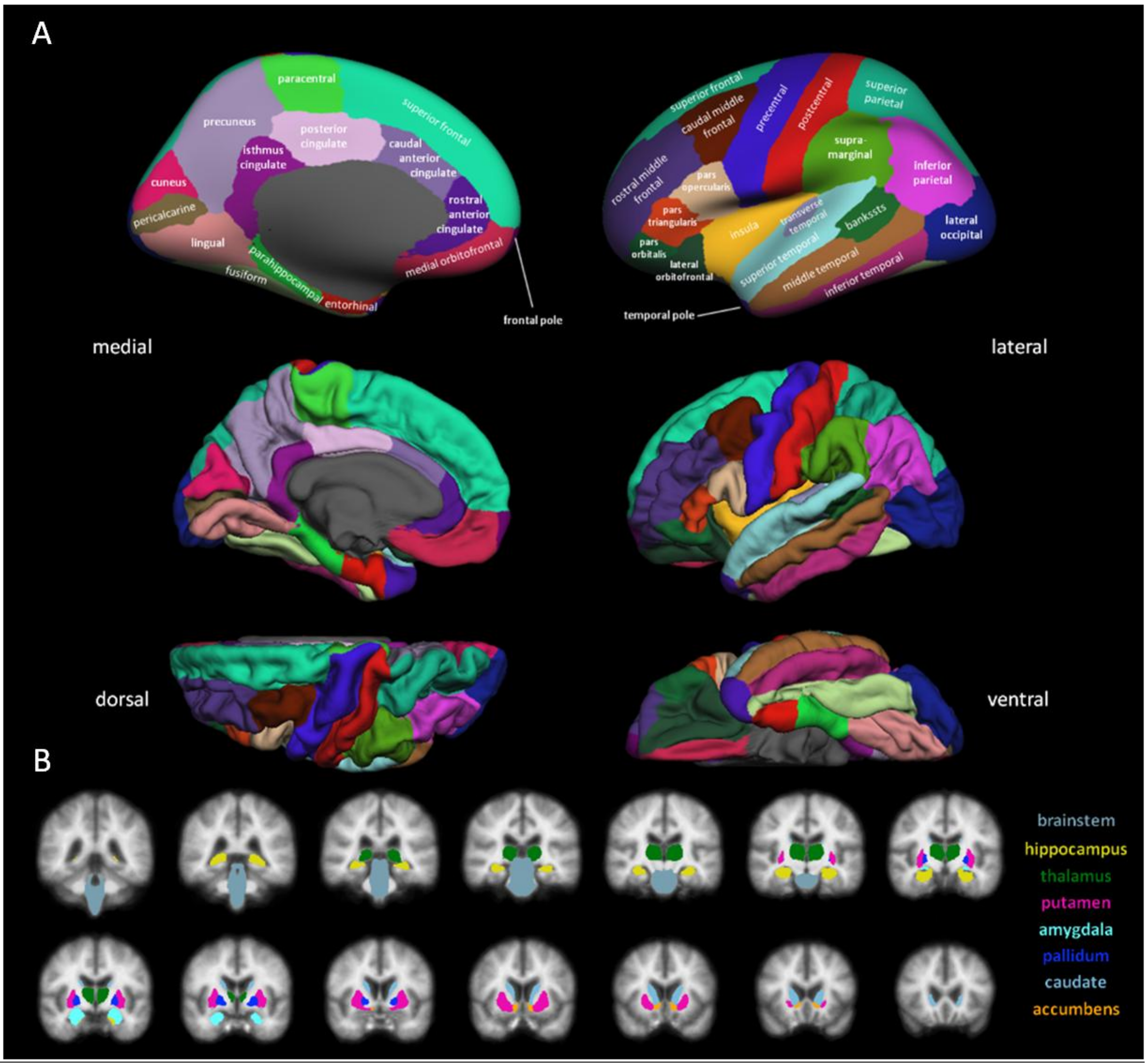

A. Top row: Illustration of all cortical labels used to define cortical nodes, overlayed on the inflated fsaverage brain. Middle and bottom rows: Same as above with labels being overlayed on the reconstructed fsaverage brain. B. Illustration of all subcortical labels used to define subcortical nodes, overlayed on the fsaverage brain. Bankssts: bank of the superior temporal sulcus

Supplementary Figure 2. Global graph measures and small-worldness plotted as a function of connectivity matrix densities.

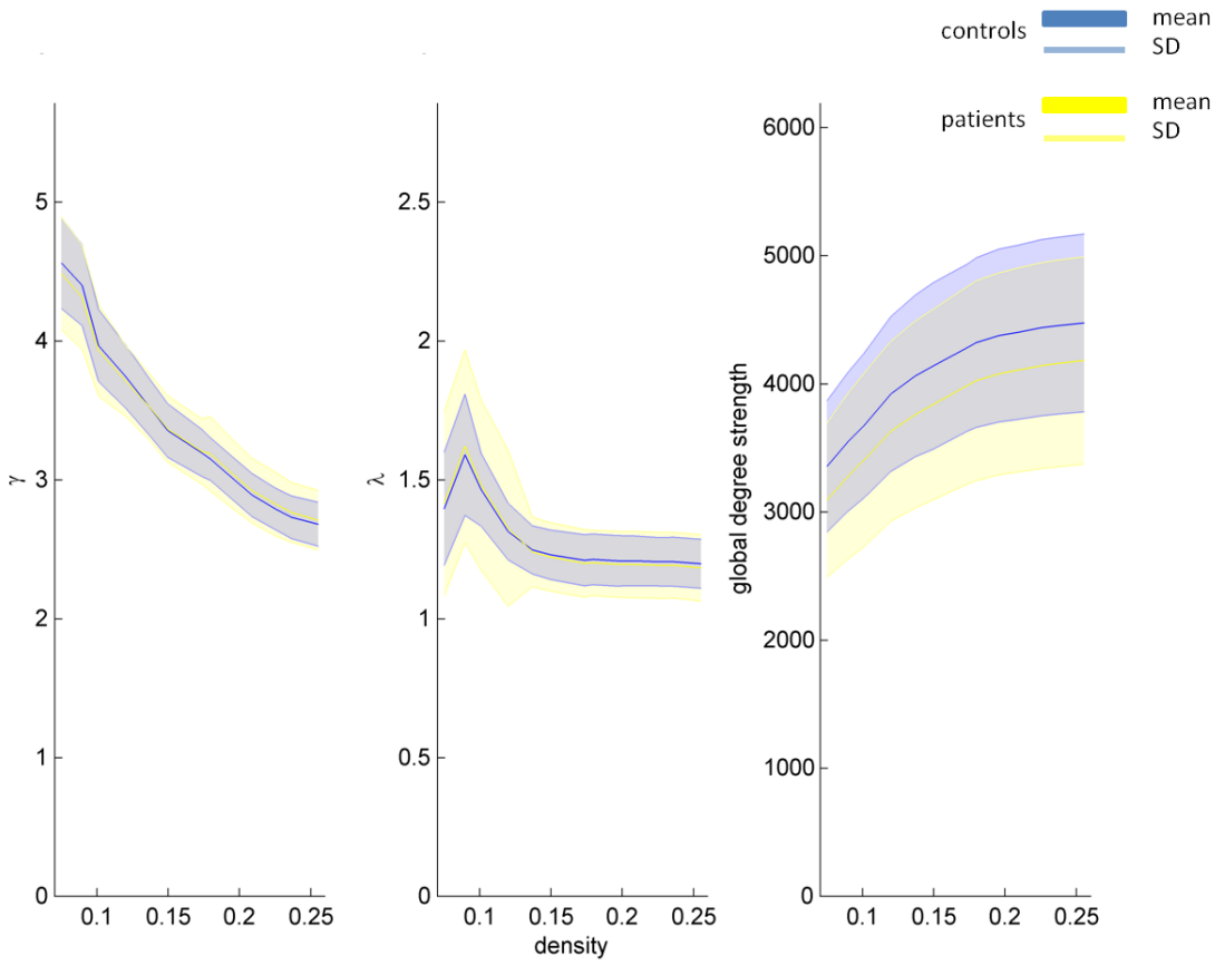

Supplement: Supplementary Information [file tp2016163x1.pdf]
